# The Lysine Methyltransferase G9a in Immune Cell Differentiation and Function

**DOI:** 10.3389/fimmu.2017.00429

**Published:** 2017-04-11

**Authors:** Sebastian Scheer, Colby Zaph

**Affiliations:** ^1^Infection and Immunity Program, Department of Biochemistry and Molecular Biology, Biomedicine Discovery Institute, Monash University, Clayton, VIC, Australia

**Keywords:** G9a, T cells, innate lymphoid cells, epigenetics, immunological memory, mouse models, infection, inflammation

## Abstract

G9a (KMT1C, EHMT2) is a lysine methyltransferase (KMT) whose primary function is to di-methylate lysine 9 of histone H3 (H3K9me2). G9a-dependent H3K9me2 is associated with gene silencing and acts primarily through the recruitment of H3K9me2-binding proteins that prevent transcriptional activation. Gene repression via G9a-dependent H3K9me2 is critically required in embryonic stem (ES) cells for the development of cellular lineages by repressing expression of pluripotency factors. In the immune system, lymphoid cells such as T cells and innate lymphoid cells (ILCs) can differentiate from a naïve state into one of several effector lineages that require both activating and repressive mechanisms to maintain the correct gene expression program. Furthermore, the long-term immunity to re-infection is mediated by memory T cells, which also require specific gene expression and repression to maintain a quiescent state. In this review, we examine the molecular machinery of G9a-dependent functions, address the role of G9a in lymphoid cell differentiation and function, and identify potential functions of T cells and ILCs that may be controlled by G9a. Together, this review will highlight the dynamic nature of G9a-dependent H3K9me2 in the immune system and shed light on the nature of repressive epigenetic modifications in cellular lineage choice.

The mammalian immune system is made up of a large number of cell types that have the ability to respond to external environmental cues and adopt a wide variety of cell fates. These lineage decisions are critical for the development of proper immune responses to pathogens as well as important for the resolution of inflammatory responses. Despite the importance of these cell fate decisions, the molecular mechanisms that control them are still not completely described nor understood. In addition to expression of cell lineage-specific master regulatory transcription factors (TFs) (1), the epigenetic landscape of the chromatin is emerging as a central control point in cellular lineage differentiation.

In the immune system, lymphoid cells such as B cells, T cells, and innate lymphoid cells (ILCs) have the capacity to respond to the external environment by modulating the expression of lineage-specific factors that are critical for protective immunity to a wide variety of pathogens. For example, the description of CD4 T helper (Th) cell subsets by Mosmann and Coffman in 1986 has provided a fundamental framework for the division of labor mediated by lymphoid cells (2). Naive Th cells respond to signals from innate immune cells—primarily secreted cytokines—to differentiate into one of several ‘lineages’ that differ in the expression of TFs, cytokines, and cell surface molecules (3). For example, Th1 cells differentiate from naive Th cells in the presence of IL-12, express the TFs STAT4 and T-bet, leading to the production of IFN-γ. In contrast, IL-4 signaling promotes STAT6 and GATA3 expression in Th cells, resulting in IL-4- and IL-13-producing Th2 cells. In recent years, additional Th cell subsets have been identified that derive from a naïve precursor cell, which include Th17 cells (that express the TF RORγt and secrete IL-17A) and Treg cells (that express the TF FOXP3 and secrete TGF-β). More recently, ILCs that have similar patterns of differentiation and gene expression have been identified. Although the differentiation of ILCs appears to occur earlier than Th cells with ‘committed’ progenitors exiting the bone marrow (4), distinct ILC subsets that are closely related to Th1, Th2, and Th17 cells (ILC1s, ILC2s, and ILC3s, respectively) have been described. Strikingly, the TF expression patterns are highly conserved between Th cells and ILCs, suggesting that generalized molecular mechanisms control lymphoid cell differentiation and function. Understanding the molecular mechanisms of immune cell differentiation will provide the basis for development of new therapies to promote immunity to infection as well as prevent inflammatory diseases caused by dysregulated immune responses.

## Epigenetic Regulation of Gene Expression

Epigenetic regulation encapsulates a wide range of mechanisms that can result in heritable changes in gene expression. From physical localization of genes within the nucleus to post-translational modifications of DNA and histones, epigenetic mechanisms can profoundly influence gene expression and alter cellular lineage development and function. The site-specific methylation and demethylation of CpG motifs in DNA by DNA methyltransferases (DNMTs) and the TET family of proteins is perhaps the best studied epigenetic mechanism that directly regulates gene expression (5). Although DNA methylation has been implicated in lymphoid cell responses (6–8), this review will focus on the posttranslational methylation of histones.

Regulation of gene expression by histone modifying enzymes is an important mechanism that regulates cellular development and differentiation. Histones can be modified posttranslationally by phosphorylation, acetylation, ubiquitination, sumoylation, and methylation (9). In particular, methylation of histone lysine residues is an important regulator of gene expression. Mixed lineage leukemia-1 (MLL1)-dependent H3K4me3 (10) and enhancer of zeste homolog-2 (EZH2)-dependent H3K27me3 (11) are the best known modifications and are associated with gene expression and repression, respectively (12–14). Other histone-methylation sites have been shown to be critical as well, including H3K9, with G9a-dependent H3K9me2 and Suv39h1/2-mediated H3K9me3 playing important roles in cell differentiation and function (15–18). Of these, H3K9me2 has been shown to modify euchromatin and dynamically regulate gene expression in differentiating cells.

In embryonic stem (ES) cells, it has been proposed that H3K9me2 marks increase across the genome as cells differentiate and acquire lineage specificity (19) although this is contentious (20). Specifically, H3K9me2 is found enriched at lineage non-specific genes, suggesting that acquisition of H3K9me2 is critical for gene silencing during differentiation (21). However, there is very little known about the role of G9a in cells of the immune system. In lymphoid cells such as T cells and ILCs, it is clear that G9a-dependent H3K9me2 is critical for cellular differentiation and function, although the mechanisms differ from ES cells. In this review, we focus on the role of the histone lysine methyltransferase G9a in lymphoid cell responses in health and disease.

## G9a is Required for Dimethylation of H3K9

G9a (*Ehmt2*) was first identified as a gene located in the major histocompatibility complex (MHC) locus in mice and human leukocyte antigen (HLA) locus in humans and was also called HLA-B-associated transcript 8 (BAT8) (22–25). The *Ehmt2* gene is located in the ~700 kb (mouse)/~1.1 Mb (human) MHC/HLA Class III region that contains over 60 genes (26) including cytokines (TNF-α and TNF-β), complement proteins C2 and C4, heat shock proteins (HSP70), and enzymes (steroid 21-hydroxylase Cyp21). The *Ehmt2* gene is made up of 28 exons that code for a 1,263 amino acid protein. A splice form lacking exon 10 that codes for 34 amino acids has also been identified, although the functional significance is still unknown (24).

The related protein G9a-like protein (GLP, EHMT1) whose gene is not located in the MHC/HLA locus forms a heterodimer with G9a *in vivo* and is critically required for the H3K9me2 methylation activity (27). Genetic deletion of either protein results in a significant reduction in H3K9me2, suggesting that both subunits are essential to the enzymatic activity (28). Mutation of the active sites has shown that the methyltransferase activity of G9a plays a larger role in H3K9me2 methylation *in vivo* (29). However, global gene expression analysis of neurons of mice with targeted deletions of either G9a or GLP identified differences that may be due to differential requirement of each subunit in gene-specific expression (30). Further, loss of GLP is associated with Kleefstra Syndrome, a rare genetic disease that is characterized by intellectual disability and other social and physical impairments (31). There has been no analysis of the function of immune cells in Kleefstra Syndrome patients. Thus, although GLP may play a specific role in regulation of gene expression, it remains to be directly tested.

**Figure 1:**
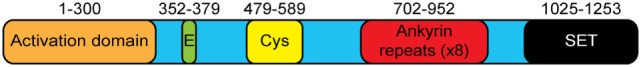
**The structure of G9a**. G9a is a 1,253 amino acid protein that has several distinct domains including an N-terminal activation domain, glutamate-rich (23 consecutive Glu residues) and cysteine-rich regions of unknown function, eight ankyrin repeat units (binding of dimethylated lysine residues), and a C-terminal enzymatic SET domain.

G9a is a 1,263 amino acid protein with several distinct domains (Figure 1). G9a does not contain a DNA-binding domain and must rely on cofactors for its localization to specific genes. Functionally, the C-terminal SET domain contains the lysine methyltransferase activity that defines the major function of this family of proteins. The SET domain of G9a is able to mono- and dimethylate H3K9 but is less efficient in mediating trimethylation (32). Consistent with this, deletion of G9a leads to a global reduction in H3K9me2 while H3K9me3 is largely unaffected (18, 28). Unlike other members of the SET domain family, G9a also has unique domains that provide additional functions. First, G9a has a series of eight 33-amino acid repeats that have homology to the ankyrin repeat domain of *Drosophila* Notch (25). This region was further shown to act as a domain that could specifically bind to dimethylated lysine residues, providing a protein that can not only generate a specific posttranslational modification but also bind to that modification (33). Interestingly, although the ankyrin repeats of G9a have an affinity for H3K9me2, GLP binds to H3K9me1 with higher affinity (33) and mice that carry a knock in of G9a with non-functional ankyrin repeats develop normally while mice with a mutated GLP have severe developmental defects resulting in perinatal lethality (34). These results further demonstrate that G9a and GLP have some non-overlapping roles *in vivo*. G9a also has a stretch of 25 glutamic acid residues as well as a Cysteine-rich region, whose functions remain unknown.

Although G9a has predominantly been studied in the context of gene repression via its methyltransferase activity on histones, it is clear that G9a also has a role in gene activation under certain conditions (35–37), which is methyltransferase-independent (discussed below). This function has been mapped to the N-terminus of the protein as the first 280 amino acids are sufficient to promote gene expression by acting as a scaffold to recruit transcriptional coactivators such as CARM1 and p300 (36, 38). Thus, G9a is a complex protein that is involved in gene repression and activation through distinct mechanisms.

## G9a is the Major H3K9 Dimethyltransferase

G9a is the enzyme that is responsible for the dimethylation of H3K9, a hallmark of silenced euchromatin (18, 28, 39–41). H3K9me2 acts as a binding site for heterochromatin protein 1 (HP1) that recruits transcriptional repressors to prevent gene activation (42). Although H3K9me2 is the main product of G9a-dependent methylation, the G9a/GLP complex has also been described to methylate H1 (43, 44) and contributes to the methylation of H3K27 (39, 45). In addition, G9a has been shown to have activity against several non-histone proteins including itself (46) though the most well-studied aspect of G9a biology is the H3K9me2-dependent repression of gene expression. From genetic and biochemical studies, it is clear that G9a-dependent H3K9me2 is associated with genomic regions that are expressed at low levels (21) but the mechanisms that regulate the dynamic methylation patterns mediated by G9a still remain unclear. Indeed, as G9a lacks a domain that would promote direct interaction with DNA or chromatin, G9a has to rely on the DNA-binding capacity of its interaction partners.

In ES cells, G9a-dependent H3K9me2 is linked to *de novo* DNA methylation (47, 48). DNA methylation of endogenous retroelements, and a subset of non-repetitive sequences including CpG-rich promoters, is reduced in G9a-deficient cells, and Dnmt3a recruitment to retrotransposons is decreased in these cells. However, the interaction between G9a, H3K9me2, and DNMTs was absent in differentiated cells (48), suggesting that functional G9a–DNMT interactions are not maintained past development. Related to this function of G9a, the sustained silencing of pluripotency-associated genes in G9a-deficient ES cells is impaired and results in the reversal from the differentiated into a pluripotent state in a significant fraction of cells (49, 50). This effect has been exploited in the generation of induced pluripotent stem cells as inclusion of a G9a-specific chemical inhibitor BIX-01298 can replace viral transduction of Sox2 in fibroblasts (51).

Taken together, a general theme of G9a playing a role in the epigenetic silencing of cell-type inappropriate genes has emerged from studies in ES cells (52, 53). However, the role of G9a in immune cell function is less well understood.

## G9a Can Methylate Non-Histone Proteins

In addition to its role as a histone lysine methyltransferase, several studies have shown that G9a is also able to methylate a wide range of non-histone targets, including G9a itself (46), CDYL1, WIZ and ACINUS, C/EBPβ, CSB, histone deacetylase (HDAC)1, mAM, KLF12, SIRT1, Reptin, MyoD, p21, and p53 (54). Although the precise role of posttranslational methylation on protein function remains unclear, methylation of non-histone proteins may affect protein stability, protein–protein interactions, subcellular localization, or function (55). Nevertheless, the precise physiological role of G9a-dependent methylation of non-histone proteins remains unclear and will not be discussed in depth in this review.

## G9a has Methyltransferase-Independent Activities

Apart from its ability to methylate substrates, G9a has also been shown to have methyltransferase-independent activities through the N-terminal domain of the G9a protein (35, 36, 38). Work from the Stallcup group was the first to show that in contrast to expectations, G9a was a strong coactivator of nuclear hormone receptor activity including androgen, estrogen, and glucocorticoid receptors by associating with the transcriptional coactivators GRIP1, CARM1, and p300 (35), and in the case of estrogen, with the receptor itself (36). G9a has also been shown to positively regulate gene expression at the β-globin gene locus (37). Regulation of the β-globin gene locus is a well-characterized system to examine the role of locus control regions (LCRs) in tissue- and stage-specific expression of genes (56). In red blood cells, the β-globin locus is regulated partially through the addition and removal of histone modifications including H3K4me3 and H3K9me2 (57). Knockdown of G9a in adult erythroid progenitor cells led to the heightened mis-expression of fetal β-globin as well as a significant reduction in adult β-globin gene expression (37), further demonstrating inhibitory and activating effects of G9a. Similar to studies with nuclear hormone receptors, the activation of gene expression by G9a was independent of methyltransferase activity. Importantly, the β-globin gene locus is regulated in a similar manner to the type 2 response *Il4-Il5-Il13* locus (58) and may provide a common mechanistic link to regulate gene expression. Thus, in addition to its repressive functions, it is clear that G9a can positively influence gene expression at select genetic loci.

## G9a in the Immune System

The vast majority of studies on the function of G9a have been carried out in ES cells and very little is known about the role of G9a in innate and adaptive immune cells. However, the ability of immune cells to respond to the external environment and differentiate into functionally distinct cell lineages is reminiscent of the cellular plasticity of ES cells and suggests that epigenetic mechanisms may be a conserved regulatory mechanism in these cell types.

In innate immune cells such as macrophages, G9a-dependent H3K9me2 has been associated with gene repression during endotoxin tolerance (59–61). Macrophages that are chronically stimulated with lipopolysaccharide (LPS) become unresponsive to further LPS stimulation through the acquisition of H3K9me2 at repressed genetic loci (61). In tolerized macrophages, G9a has been shown to interact with the TF ATF7 as well as several members of the NF-κB family including RelB, RelA, c-Rel, and NF-κB1 (59–61). It is proposed that G9a is recruited to specific loci by these factors to deposit H3K9me2, leading to gene repression. However, a direct role for G9a in macrophages during endotoxin tolerance has not been tested. Nevertheless, these studies identify a role for G9a in gene silencing during cellular responses to inflammatory signals. Consistent with this role in promoting tolerance, G9a has been also shown to limit JAK/STAT signaling in *Drosophila* following viral infections (62). In the absence of G9a, viral infection leads to increased lethality in flies but is not due to increased pathogen burden but due to heightened expression of JAK/STAT-dependent target genes. Thus, G9a is an important regulator of innate inflammatory gene expression.

G9a has also been implicated in several aspects of T cell biology. Although genome-wide studies mapping the binding of G9a or the deposition of H3K9me2 in immune cells has not been carried out due to technical reasons, a descriptive genome-wide analysis of H3K9me2 marks in resting human lymphocytes using ChIP-on-chip methods demonstrated that this epigenetic mark is enriched on genes that are associated with several specific pathways including T cell receptor signaling, IL-4 signaling, and GATA3 transcription (63). Furthermore, lymphocytes isolated from patients with type I diabetes displayed a distinct H3K9me2 profile, with genetic regions that had increased (CXCL3, CTLA-4, SLC17A4) and reduced (RARA, CAMK4, TNF) levels of H3K9me2 (64). Thus, G9a-mediated H3K9me2-dependent regulation of T cell responses may be associated with T cell function as well as development of inflammatory diseases such as diabetes.

More recently, cell lineage-specific deletion of G9a has been used to delineate the role of G9a in immune cells. Three independent strains of mice with a “floxed” G9a allele (*G9a*^fl/fl^ mice) have been generated (16, 65, 66). Crossing *G9a*^fl/fl^ mice with *Cd4*-Cre mice or *Lck*-Cre mice results in a T cell-specific deletion of G9a (*G9a*^ΔT^ mice). *G9a*^ΔT^ mice are born and develop normally and have no discernable defects in the generation of T cells in the thymus, spleen, or lymph nodes (16, 67), suggesting that unlike ES cells G9a is dispensable for cellular development of peripheral naive T cells. However, upon activation of T cells *in vitro* or *in vivo*, G9a was shown to play a critical role in regulating the function of Th cells. Consistent with the ability of G9a to promote and repress gene expression, G9a-deficient Th cells had a failure to produce certain cytokines while overproducing others (15, 16). Under distinct differentiating conditions, G9a was differentially required to activate or repress specific gene programs.

Th1 cells that produce IFN-γ are critical for immunity against intracellular pathogens including bacteria, viruses, and protozoan parasites (68). Strikingly, the absence of G9a in T cells has no effect on the development or magnitude of Th1 cell responses *in vitro* or *in vivo* (16). There was no difference in the frequency of Th1 cells that developed following activation of G9a-deficient T cells or wild type T cells in the presence of a G9a-specific inhibitor under Th1 cell-promoting conditions (16). Thus, from our understanding so far, G9a is dispensable for Th1 cell responses.

Immunity to infection with parasitic helminths such as the whipworm *Trichuris muris* is associated with a polarized type 2 cytokine response, with the production of IL-4, IL-5, and IL-13 by Th cells leading to mucus production, intestinal epithelial cell turnover, and worm expulsion (69). Infection of *G9a*^ΔT^ mice with *Trichuris* resulted in significantly reduced frequencies of protective Th2 cells, heightened frequencies of non-protective Th1 cells, and susceptibility to infection (16). This was consistent with a reduction in the production of type II cytokines by Th cells after *in vitro* activation. A role for G9a in repressing promiscuous type I cytokine gene expression in Th2 cells was only observed temporarily, as H3K9me2 is replaced by H3K27me3 at the *Ifng* locus during differentiation (70). Thus, G9a is required for activation of the type II cytokine gene program. G9a-deficient Th2 cells express comparable levels of the major Th2 cell transcriptional factors including GATA3 and STAT6 but fail to produce type II cytokines, suggesting that G9a is a central component of the transcriptional machinery for type II cytokines. As the *Il4-Il5-Il13* locus is similar in genetic structure to the β-globin locus, it is perhaps not surprising that the ability of G9a to transactivate the type II cytokine gene locus was also independent of its methyltransferase activity (16). Taken together, these results identify a role for G9a as a critical component of the Th2 cell regulatory machinery.

In contrast to Th2 cells, G9a plays an important role in limiting Th17 and Treg cell differentiation that is dependent upon its methyltransferase activity (15). Activation of G9a-deficient Th cells under Th17- or Treg cell-promoting conditions resulted in a significant increase in the frequencies of IL-17A-producing and FOXP3-expressing cells, respectively (15). Inhibition of G9a methyltransferase activity with the small-molecule inhibitors BIX-01294 or UNC0638 also resulted in enhanced Th17 and Treg cell differentiation. Unlike the proposed role for G9a in ES cells, H3K9me2 is not deposited at lineage-promiscuous genes to control lineage differentiation in Th cells. Instead, G9a-dependent H3K9me2 is found at high levels in naive undifferentiated Th cells and is rapidly lost at lineage-specific and -non-specific loci after T cell activation (15). The loss of H3K9me2 alone is insufficient to promote gene expression, providing an explanation for the lack of a phenotype in the steady state. Thus, in naive T cells, H3K9me2 and G9a act as an additional layer of negative regulation to maintain cells in a naive state. Mechanistically, loss of G9a-dependent H3K9me2 results in an increase in the accessibility of the chromatin to transactivating factors, which leads to heightened responsiveness to external signals such as cytokines. In the case of Th17 and Treg cells, G9a-deficient Th cells are ~40 times more sensitive to TGF-β (15). Taken together, these results suggest that G9a, through the deposition of H3K9me2 at a wide variety of immune genes, is specifically important in naive T cells to repress gene expression, possibly by limiting accessibility of TFs and coactivators to specific genetic loci.

Unlike naive T cells, G9a has been proposed to repress expression of CD25 and CD27, cell surface receptors that are associated with cell activation and proliferation, in memory T cells during viral infection (71). Through a specific interaction with the transcriptional repressor Blimp-1 (PRDM1), G9a (but not EZH2) is recruited to specific genetic loci resulting in gene repression. In the absence of Blimp-1, reduced levels of H3K9me2, H3K9me3, and H3K27me3 is observed, suggesting that G9a is critical for the silencing of genes during memory T cell differentiation and development. Whether G9a is required for memory T cell generation has not been examined directly; however, this is an active area of research, which will shed further light on the role of G9a in T cell memory development. Thus, in the absence of G9a, other repressive modifications such as Suv39h1/2-dependent H3K9me3 and EZH2-dependent H3K27me3 may compensate. Although, the details remain unclear, the identification of the epigenetic mechanisms in memory T cell development and function will be critical for the optimal design of vaccines as well as the development of therapeutic strategies to target dysregulated memory T cell responses in inflammatory diseases.

In addition to T cells, G9a also plays a critical role in the development and differentiation of ILCs (72). Mice with a hematopoietic cell-specific deletion of G9a (generated by crossing *G9a*^fl/fl^ mice with *Vav*-Cre mice) have reduced numbers of group 2 ILCs (ILC2s) and increased frequencies of ILC3s in all tissues examined. Although the small number of ILC2s present in lung tissue are phenotypically normal, they are dysfunctional, failing to produce effector cytokines IL-5 and IL-13 following stimulation with the activating cytokine IL-33 or following intranasal administration of the protease allergens papain or house dust mite antigen (72). Genome-wide expression analysis of bone marrow-derived ILC2 precursors identified a global shift in expression from ILC2-specific transcripts to ILC3-specific genes, placing G9a and H3K9me2 as a central regulator of the ILC2/ILC3 lineage choice.

In contrast to T cells and ILCs, G9a appears to play a minor role in B cell development and function (52, 67). An early study using a V–D–J minilocus suggested that G9a inhibited germline transcription and recombination (73). However, mice with a B cell-specific deletion of G9a (*Mb1*-Cre) fail to show any overt phenotypes, including displaying normal V–D–J recombination (67). The absence of G9a did result in a skewed usage of the κ light chain over the λ light chain, as well as a slight reduction in IL-4- and LPS-induced proliferation and differentiation into plasma cells. This reduction in plasma cell differentiation is consistent with a study showing that G9a directly interacts with Blimp-1, a critical plasma cell differentiation factor (74). Thus, G9a is dispensable for normal B cell development and has little effect on most functions of B cells. However, it is possible that G9a has a subtle role in specific aspects of B cell biology that remain to be determined.

Together, these results demonstrate that G9a is an important regulator of immune cell function. However, the precise mechanisms are yet to be defined. In the following sections, immune functions regulated by proteins known to interact with G9a will be discussed and potential mechanisms for G9a-dependent regulation will be proposed.

## Interactions and Potential Roles of G9a

**Figure 2:**
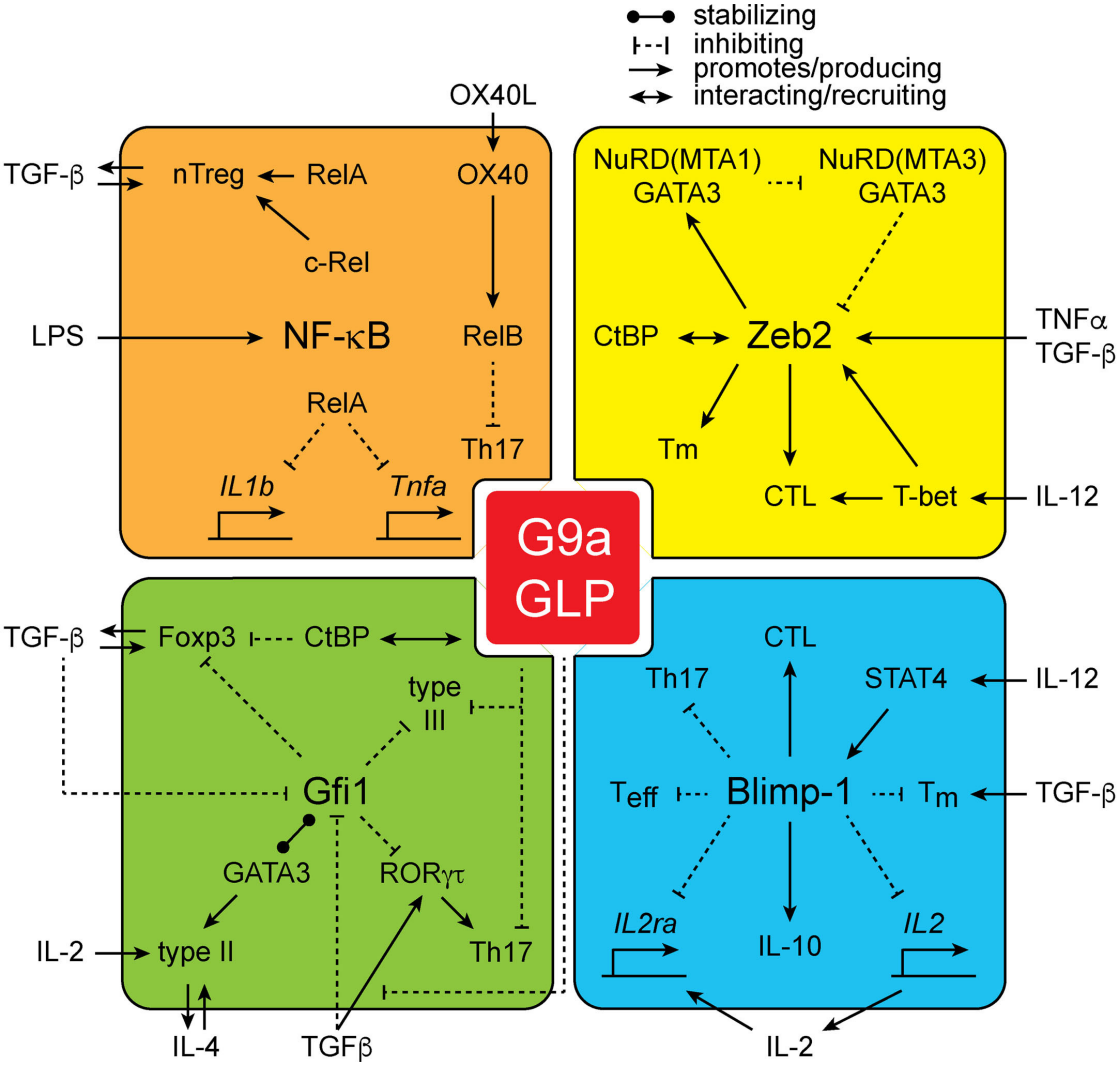
**The interactions of G9a**. G9a interacts with a large number of proteins including NF-kB family members RelA, c-Rel, and RelB, as well as zinc finger E-box-binding (Zeb2), growth factor independent 1 (Gfi1), and Blimp-1.

From the studies outlined above, it is clear that G9a is a central regulatory node in the establishment of the epigenetic landscape of cells and is critical for shaping cellular identity. However, how G9a mediates its function is poorly understood. As G9a lacks a DNA-binding domain, it is dependent upon additional cofactors for its localization at specific genetic loci. Several classes of G9a-interacting proteins have been identified (Figure 2). Strikingly, many of the G9a cofactors have important roles in immune cell development and function, potentially offering a mechanistic explanation for the phenotypes associated with loss of G9a in lymphoid cells.

### Growth Factor Independent 1

The transcriptional repressor growth factor independent 1 (Gfi1) is an important regulator of immune cell development and function (75). Gfi1 is able to bind to a large number of promoters and enhancers (76) and plays a central role in gene silencing through the recruitment of repressive modulators including histone methyltransferases, HDACs, and histone demethylases (77–79). Gfi1 has been shown to directly interact with G9a (78, 80) and Gfi1-deficient cells display a significant decrease in H3K9 methylation (78). Further, the phenotypes associated with Gfi1 deficiency in the immune system are strikingly similar to those observed in G9a-deficient mice, suggesting that Gfi1 is central to the function of G9a.

Gfi1 was first identified as a factor that, when induced or overexpressed in an IL-2-dependent T cell line, led to IL-2-independent growth (81). Furthermore, Gfi1 was shown to reduce the requirement for IL-2 by modulating the cell cycle regulation of T cells (82, 83). More recently, Gfi1 has been described to have multiple effects that echo the phenotypes seen in mice lacking G9a in T cells. First, Gfi1 is critical for promoting the differentiation of Th2 cells through a variety of mechanisms including increasing GATA3 stability, enhancing Th2 cell proliferation, inhibiting Th1 cell differentiation, and promoting expression of type 2 cytokines (84–87). Under Th2 cell-polarizing conditions, Gfi1 was found to be highly upregulated by IL-4 in a STAT6-dependent manner (84) and retroviral overexpression of Gfi1 in Th2 cells resulted in increased proliferation and survival (84). Gfi1-deficient T cells failed to optimally produce IL-4 after *in vitro* stimulation or following infection with the helminth parasite *Schistosoma mansoni* (87). Mechanistically, Gfi1 inhibits the proteasomal degradation of GATA3 through its N-terminal Snail/Gfi1 (SNAG) domain (85). As Th2 cell differentiation is impaired in G9a-deficient T cells (16), it is possible that G9a–Gfi1–GATA3 interactions are critical for the establishment of a transcriptional module that results in activation of the type 2 cytokine locus. Based on the role of the G9a N-terminus in activating nuclear hormone receptor-dependent gene expression by acting as scaffold (38), these results suggest that in Th2 cells, the N-terminus of G9a may aid in recruitment of Gfi1, GATA3, and potentially other factors required for optimal Th2 cell development.

Growth factor independent 1 has also been implicated in the differentiation of Th17 and Treg cells (88). Downregulation of Gfi1 expression by TGF-β is critical to allow expression of IL-17A/F in Th17 cells and CD103 in Treg cells (88) as well as surface expression of the ectonucleotidases CD39 and CD73 (89). Gfi1 potentially recruits the lysine demethylase LSD1 to these genetic loci to reduce the activating methylation marks. Upon stimulation with TGF-β, Gfi1 expression is reduced allowing optimal Th17 and Treg cell differentiation. Gfi1-deficient T cells display increased production of IL-17A and increased FOXP3 expression in response to TGF-β, which is identical to G9a-deficient T cells (15). Further, similar to the dysregulated expression of IL-17A observed in G9a-deficient Th2 cells (16), Gfi1 is required to silence IL-17A expression in Th2 cells (88). Thus, it is intriguing to hypothesize that Gfi1–G9a interactions are critical to restrain Th17 and Treg cell responses, linking transcriptional repression to epigenetic gene silencing.

Growth factor independent 1 is also an important regulator of ILC2 development and function (90). Expression of Gfi1 is correlated to the expression of the IL-33 receptor (*Il1rl1*, ST2) and GATA3. Loss of Gfi1 in ILC2s leads to impaired expression of GATA3 and an upregulation of IL-17A expression. This is reminiscent of the role of G9a in ILC biology, where G9a is required to repress ILC3-specific genes during ILC2 development (72). However, the effects of G9a and potentially Gfi1 appear to be dependent upon the methyltransferase-dependent gene repressive effects unlike in T cells.

Taken together, these results suggest that G9a–Gfi1 interactions are critical for their functions in T cells and ILCs. Future studies defining the molecular basis for these interactions may provide novel therapeutics to inhibit dysregulated Th2 cell responses that are associated with diseases such as asthma and allergy.

### Zinc Finger E-Box-Binding Protein 2

Zinc finger E-box-binding proteins (Zeb1 and Zeb2) are TFs that have primarily been associated with TGF-β-dependent epithelial–mesenchymal transition in tumor cells (91, 92). Zeb proteins repress expression of several epithelial genes such as E-cadherin through the recruitment of repressive molecules including C-terminal binding protein (CtBP) and components of the nucleosome remodeling deacetylase complex (NuRD) including HDAC1. Recently, in a proteomic screen of G9a-interacting proteins in activated and endotoxin-tolerant macrophages, G9a was found to be strongly associated with components of several complexes that regulate chromatin structure including the Swi/SNF complex, NuRD complex, and CtBP/CoREST complexes (61). In breast cancer cells, G9a was shown to be a component of a complex containing Zeb2 and the NuRD component MTA1 (93). Interestingly, this was a highly dynamic complex that switched between a GATA3/G9a/MTA3 complex with a Zeb2/G9a/MTA1 complex that cross-regulated each other. A potential complex of G9a, Zeb2, and GATA3 that interacts with the NuRD complex may provide a molecular mechanism for how G9a, independent of its methyltransferase activity, regulates type 2 gene expression in Th2 cells. It is clear that nucleosome remodeling is an important aspect of type 2 cytokine expression (94, 95); however, the precise molecular mechanisms remain elusive. In addition, a direct role for Zeb2 in Th2 cell differentiation has not been evaluated.

Zinc finger E-box-binding protein has been shown to regulate the development of protective CD8 T cells during viral infection (96, 97). Following infection with lymphocytic choriomeningitis virus (LCMV), Zeb2 expression is induced in effector CD8 T cells that express the surface marker KLRG1 and produce IFN-γ. The upregulation of Zeb2 is dependent upon the TF T-bet (*Tbx21*) and expression of Zeb2 is critical for the terminal differentiation of effector CD8 T cells that are required for immunity to viral infection. As GATA3 has been implicated in CD8 T cell function (98, 99), it is possible that a Zeb2/G9a/GATA3 complex is critical for the function of CD8 T cell function during viral infection. Further, as TGF-β has been shown to play an important role in shaping an effective CD8 T cell response and memory formation (100–103), and G9a is critical for regulating TGF-β responsiveness (15), the intersection of these pathways may prove important for defining the molecular mechanisms of T cell biology during infection.

### NF-κB

The NF-κB family of TFs plays a central and critical role in all aspects of immune cell biology (104). Both, the canonical (c-Rel, p65/RelA, and p50/NF-κB1) and non-canonical (RelB and NF-κB2) family members have been shown to be important regulators of immune cell function (105, 106). In a proteomic screen for G9a-binding partners, it was found that several members of the NF-κB family (RelB, c-Rel, RelA, and NF-κB1) are highly enriched for binding to G9a in macrophages (61), and RelB had been previously shown to associate with G9a during endotoxin tolerance, which was associated with gene silencing (59). In addition, the ankyrin repeats of GLP have been shown to directly recognize and bind to a monomethylated lysine residue in RelA, which directly links the G9a/GLP complex to attenuate cell proliferation and inflammatory responses in immunologically important genes such as *Il1b* and *Tnfa* (107). Together, these studies suggest that induction of NF-κB by LPS results in the recruitment of G9a and gene silencing, leading to endotoxin tolerance. Thus, under these circumstances, G9a-dependent dimethylation of H3K9 is important for gene repression during cellular activation.

In T cells, NF-κB family members have been shown to be important for Treg cell development and function (108–113) as well as implicated in Th17 cell responses (114, 115). In the absence of c-Rel or RelA, thymic-derived natural Treg (nTreg) cells have a severe developmental defect and show reduced ability to suppress inflammatory responses. In contrast, the absence of G9a had no effect on nTreg cell development or function and the development of peripherally-activated Treg (pTreg) cells was enhanced in the absence of G9a (15). Thus, whether G9a/NF-κB interactions are required for Treg cell function is unclear.

However, the interaction between G9a and the non-canonical family member RelB may prove to be more important in T cell biology. Recently, RelB was shown to have an important function in limiting the development of Th17 cells (114). Costimulation of Th17 cells through OX40–OX40L interactions resulted in a significant reduction in IL-17A expression. This effect was mediated by RelB-dependent recruitment of G9a to the *Il17a* locus, resulting in repression of *Il17a* expression. Further, in the absence of RelB, Th17 cells show reduced pathogenicity and inflammation in experimental autoimmune encephalomyelitis (EAE) (114). Thus, it is likely that RelB/G9a interactions, possibly downstream of OX40/OX40L signaling, are required for optimal development of Th17 cells.

It is clear that there is a significant interaction between NF-κB and G9a, although the precise molecular mechanisms in distinct immune cells are yet to be elucidated. Nevertheless, placing G9a in the context of NF-kB signaling suggests that there is a close relationship between inflammatory signal transduction pathways and the epigenetic machinery to control gene expression during an immune response.

### Blimp-1

Blimp-1 (*Prdm1*) is a zinc finger protein which directly interacts with G9a and has been shown to be central to the development of immune responses. Blimp-1 is a transcriptional repressor that contains a non-catalytic PR domain that is related to the SET methyltransferase domain, and a zinc-finger DNA-binding domain (74). In the absence of Blimp-1, mice develop a lethal multiorgan inflammatory disease caused by an accumulation of effector and memory T cells (116), thus making Blimp-1 an important regulator of the adaptive immune response. Blimp-1 is also required for repression of *Il2* transcription in T cells, resulting in an autoregulatory loop that controls immune responses (117, 118). In addition, Blimp-1 is crucial for the IL-27-dependent induction of IL-10 by Th1 cells (so-called Tr1 cells) following infection with *Toxoplasma gondii* or influenza A virus (119, 120). Moreover, Blimp-1 is critical for the development of terminally differentiated effector CD8 T cells (121) and controls the development of “exhausted” CD8 T cells during chronic viral infection (122). Thus, Blimp-1 plays an important role in T cell-mediated immunity to infection.

In CD8 T cells, Blimp-1 has been directly linked to G9a-dependent H3K9me2-mediated repression (71). Following viral infection, Blimp-1 recruits G9a to repress expression of CD25 and CD27 and limit the expansion and proliferation of CD8 T cells. However, in addition to H3K9me2, G9a was also associated with increased levels of H3K9me3 and H3K27me3, modifications that are not mediated by G9a, suggesting that additional methyltransferases were associated with Blimp-1 and involved in gene silencing during effector differentiation. Thus, in addition to G9a, other mechanisms are likely to be required for the repression of lineage non-specific genes in differentiating cells.

In Th cells, similar to results in *G9a*^ΔT^ mice (15), mice with a T cell-specific deletion of Blimp-1 have increased frequencies of Th17 cells in the intestinal mucosa (123). Furthermore, IL-23-induced Blimp-1 has been shown to be critical for the development of inflammatory Th17 cells that are required for the pathogenesis of EAE (124). Thus, mice lacking Blimp-1 in T cells fail to develop EAE. Although it remains unknown whether Blimp-1 and G9a directly interact in CD4 T cells, it is not outrageous to suggest that G9a/Blimp-1 interactions may be critical for the development of pathogenic Th17 cells, and blockade of this interaction may provide a new therapeutic strategy to prevent inflammatory diseases associated with dysregulated Th17 cell responses.

## Inhibition of G9a Activity by Chemical Probes

Currently, there are several chemical probes that specifically target the methyltransferase activity of G9a. BIX-01294 was first identified in a high-throughput screen (125) and was shown to bind to the SET domain of G9a and GLP in the peptide-binding site, preventing methylation (126). BIX-01294 was subsequently optimized through structure–activity relationships to generate UNC0224, UNC0321, and E72 that showed increased activity and specificity (127, 128). The further development of UNC0638 and UNC0642 resulted in a potent, specific, stable, and cell-permeable inhibitor of G9a (129). However, UNC0642 has poor pharmacokinetics for *in vivo* use. More recently, an additional inhibitor of G9a that is unrelated to UNC0642, called A-366, was discovered through an independent high-throughput screen (130, 131). Treatment of mice with A-366 showed no overt toxicity and was able to reduce the growth of tumor xenografts (131). Thus, although A-366 has not been tested in the context of inflammatory disease, these results suggest that inhibition of G9a could prove to be a significant therapeutic strategy to modulate immune responses.

## Concluding Remarks

It is clear that G9a is a central control point in lymphoid cell development, differentiation, and function. Acting through its diverse binding partners, G9a can repress and activate gene programs associated with a wide variety of immune responses. As G9a has been shown to be amenable to drug inhibition, blocking the function of G9a may provide a new therapeutic modality to modulate a wide variety of inflammatory diseases. For example, both Blimp-1 and G9a are required to limit Th17 cell development in a cell-intrinsic manner (15, 123). However, the increased frequency of Th17 cells does not result in enhanced pathogenicity of inflammatory diseases such as EAE or intestinal inflammation (15, 124), demonstrating that reducing the activity of Blimp-1 or G9a, or by inhibiting their interaction, may be a viable method to reduce the development of pathogenic Th17 cells. Future studies will define the precise role of G9a in immune cell development, differentiation, and function and determine the relevance of G9a as a drug target to treat inflammatory disease.

## Author Contributions

All authors listed have made substantial, direct, and intellectual contribution to the work and approved it for publication.

## Conflict of Interest Statement

The authors declare that the research was conducted in the absence of any commercial or financial relationships that could be construed as a potential conflict of interest.
